# Stress and its impact on elite athletes' wellbeing and mental health—a mini narrative review

**DOI:** 10.3389/fspor.2025.1630784

**Published:** 2025-07-16

**Authors:** Barbara Nuetzel

**Affiliations:** Department of Psychology, Deutsche Hochschule für Prävention und Gesundheitsmanagement, Saarbrücken, Germany

**Keywords:** stress, wellbeing, mental health, resources, elite athletes, elite sports

## Abstract

Within the context of elite sports, the term “health” is tantamount to maximum physical performance. From the very beginning of their athletic careers, elite athletes are trained to prioritize performance over health in order to be successful. To reach the top and competitive goals, they often cannot avoid situations that might be endangering their health. A career in elite sports means that elite athletes must take extreme health risks and at the same time protect their health to continuously deliver peak performances. According to Fletcher and Hanton, psychological research is confronted with a particular challenge because elite athletes are subject to numerous stressors both during and after their sports career. The examination of the relevant stress-related factors (relationships, competitive pressure, defeat, injuries, etc.) points to a growing body of evidence signifying that these factors could be responsible for mental health problems in the target population. Because these challenges are multidimensional in nature, a comprehensive understanding of this topic's complexity is required. To obtain an understanding of stressors and resources in elite sport, the author performed a narrative mini review of 3 databases covering studies published up to 2023.

## Introduction

According to the Medical and Scientific Commission of the International Olympic Committee (IOC), the protection of an athlete's health is even a primary goal. As reported by the Olympic Charter, the International Olympic Committee (IOC) and the International Sports Federations (IFs) have an obligation among others to encourage and support measures protecting the health of athletes (7).

### Definitions

Mental health is not only the absence of mental illness but also mental wellbeing and the ability to fully realize one's potential [c.f. (8, 9)]. Remaining mentally healthy is also a prerequisite of top-performance in the context of elite sports [(10); c.f. (11, 12)].

However, the term “health” within the context of elite sports is mainly associated with highest performance at top level. To be successful in competitions and to reach their performance goals, elite athletes often cannot avoid dangerous or risky situations (2).

In this sense, elite sport is characterized by a health paradox: On the one hand, athletes are obliged to maintain their health in order to continue to perform at their best; on the other hand, they must constantly risk their health by not only pushing themselves to their physical and mental limits, but also—if necessary—training and competing in pain or injury (3, 13). The necessity to overstep limits over and over again causes continuous stress and can result—as a consequence—in a physical and mental overload.

Stressor-induced overload is not necessarily visible for the athletes' environment. Neglecting, ignoring or trivializing states of overload is a typical characteristic of the so-called “culture of risk” (14) in elite sport. In this sense, the developmental process of mental health problems is often kept secret, because developing mental problems is frequently equated with weakness and incapacity to deal with pressure in elite sport.

As the exposure to stress consistently confronts the athlete to deal with common stressors of life as well as with sport-specific stressors, which are seen as potential risk factors for changes of mental health, staying physically and mentally healthy represents a huge challenge in the sense of coping successfully with the state of tension created by stressors in order to generate and to protect mental health (11).

For this reason, stressors have been the focus of many studies for some time now, which deal with stress-triggering factors in elite sport (15, 16).

Various studies have already shown that elite athletes experience numerous stressors within the context of their sports (4, 17–19). Athletes are faced with continuous pressure to perform, participation in competitions, high demands from associations, sponsors, coaches and society in general and they are also confronted with individual, personal stress triggers, such as perfectionism (20).

### Mental health issues in elite sport

Based on current data, there is reason to believe that mental health issues of elite athletes need to be viewed as the answer to being continuously exposed to different stressors in elite sports. On the one hand, scientific research could show over the past few years that elite athletes, just like the general populace, are not immune to mental health issues (1, 12, 21, 22). Athletes are in fact at least as susceptible to ill effects on their mental health as the general populace (19, 23, 59). On the other hand, recent studies on mental health in elite sports assume an association of both sports-specific and general stressors with mental ill-health of elite athletes [e.g., (24)].

Although health research in elite sport suggests that athletes are exposed to many physical, psychological and social stressors there is surprisingly little evident knowledge about the role of perceived stress when it comes to the mental health of elite athletes (19). One of the main reasons for this research gap lies in causality problems, more precisely in the difficulty to exactly establish the point when a stressor becomes a stressful experience for athletes, which exceeds their coping skills and thus jeopardizes their mental health, wellbeing and resilience (25).

Reardon and Factor (5) point to the fact that it is still unclear how many elite athletes suffer from mental health issues due to their high-performance sports, how many athletes would be afflicted regardless of their sports had they chosen a different profession, and how many of them take precautions (against health problems) during and after their athletic career.

Another reason why scientific research in the field of mental health in elite sports is still in its beginning stages is that most research has focused on pathology rather than prevention (21). It usually does not cover the necessity of targeted prevention and thus leads to trivialization or even denial of potential symptoms or complaints among the athletes (26). The disclosure of mental problems is often equated with weakness in this target group. According to Reardon and Factor (5), the non-disclosure of mental problems in the sense of trivialization among athletes can be induced by the fear of exclusion from the team, from competitions, or even of losing their athletic existence or identity (27).

### Conceptualization of health

This can be explained by the elite athletes' (and often also the coaches') conceptualization of health, which they base on a mechanical perspective, i.e., they see the body as a means to an end. A body must be in perfect working condition in order to produce athletic top performances during an athletic career (28). If the body is affected by injuries or other health-related issues, these need to be treated or “healed” in terms of the relevant sports-specific performance capability as quickly as possible (3). Ideally, any long-term health management in elite sports is based on an analysis of health in terms of the interdependencies between psychological, biological, and sociocultural factors (29).

Corresponding interdependencies are suspected to exist for mental health related problems in elite sports, and they seem to vary from athlete to athlete (19). Therefore, the individual biography as well as the general context of each athlete, including family, hormonal, medical, and other personality- and development-specifics need to be considered in order to reach a clear diagnosis (30).

### Protecting resources in terms of mental health

Since elite athletes are continuously confronted with the most varied environments, demands and stressors (31), it seems to be desirable especially from a salutogenetic perspective to protect their health (3, 11). For example, it has been proven that a pronounced sense of coherence positively correlates with an improved subjective perception of one's own health. It has also been observed that a negative correlation exists between a strong sense of coherence and performance affected by injury or health-related restrictions (32). These observations point to the fact that the sense of coherence can generally assumed to be a protecting resource in terms of health, even though concrete results are still to be supplied concerning long-term consistency in elite athletes (11).

Also, resistance resources such as resilience (25), self-confidence (33), and mindfulness (34) have been the focus of scientific attention in order to analyze the possibly protective effect of these resources on mental health within the context of elite sports.

As mentioned above, elite athletes are permanently required to both protect and risk their health while at the same time maintain their optimal performance capability (3).

### Aims

Taking this scenario into account, the purpose of this narrative mini review is twofold: (1) to provide an overview on stressors and resources in the context of mental health in elite sports and (2) to propose practical implications for best practice and research in mental health literacy within elite sport relying on collaboration between sports psychiatry, sport psychology, and clinical psychology. This narrative mini review is an opportunity to better understand and discuss the importance of mental health and wellbeing against the background of stressors and resources in elite sports.

## Methods

Selection of articles for this narrative mini review consisted of two steps. The author analyzed all matches based on title or abstract first. If the information contained therein did not suffice, the relevant full text was consulted, as well.

The following process was applied to identify documents relevant to this narrative mini review: (1) Search in 2 electronic databases (PubMed and PsychINFO) and (2) complementary search in Google Scholar. The following search terms were used for the search:

[“stress”] OR [“stress predictor”] OR [“stress predictors”] OR [“stress-related strain”] OR [“stress-related strains”] OR [“stress-related demand”] OR [“stress-related demands”] OR [“stress-related pressure”] OR [“stress-related pressures”] OR [“stress exposure”] OR [“stress-related tension”] OR [“stress-related tensions”] OR [“perception of stress”]

AND

[“Mental health”] OR [“mental health effect”] OR [“mental health effects”] OR [“wellbeing”]

AND

[“resources”] OR [“psychological resources”]

AND

[“Elite athlete”] OR [“elite athletes”] OR [“competitive athlete”] OR [“competitive athletes”] OR [“high-level performance athlete”] OR [“high-level performance athletes”] OR [“professional athlete”] OR [“professional athletes”]

The publishing date was not restricted covering studies published up to 2023.

## Stressors in elite sports

Typical stressors in elite sports can be categorized into sports-related and organization-related stressors (4), including poor communication, such as lacking or negative feedback (35), or the relationship among the team or with associations, organizations, and their representatives (16).

One of the major sports-related stressors is pressure to perform. It is often enforced by the athletes' worry about their potential, fear of failure based on competitions lost in the past, and conflicts with coaches and/or team members (36, 37). Hence, the fear of being excluded from the team and/or from competitions [what in consequence threatens the athletic existence, (27)] leads to non-disclosure of stress overload and mental strains among athletes [c.f. (5)].

Injuries are another essential factor in the category of sports-related stressors (38). According to Bußmann and Alfermann (39), the inability to communicate and cope with fears are a significant internal factor pertaining to injury-related stress development.

Pressure to perform in terms of not only athletic, but also intrapersonal and financial aspects is one of the stressors that is mentioned most often (40). In this regard, also fear for livelihood plays a central role for the athletes because they usually forgo a well-founded professional education due to the intensive time requirements for training and competitions (41). After their athletic career, athletes often feel unprepared for daily life. Besides the transitional time between junior and senior phases, the career end is the major stressor as it is based on a complex interconnection of different stressors (42). The career end is often perceived as stressful and mentally straining if it occurred involuntarily, for example, due to an injury. According to Anshel and Wells (40), the transitional time between junior and senior phases is experienced as particularly stressful, as well, because of the simultaneous demanding phases in school and job with many challenges both in athletic and non-athletic areas (43).

Furthermore, the typical concept of health in elite sport is not beneficial for identifying mental health issues not least because of its primary focus on biomedical and functional aspects. Due to the primary focus on the body as a means to an end with regard to the production of athletic top performances (28), health issues are mainly treated bio-medically in the sense of repairing damage on the basis of a medical diagnosis (3).

Current research results (44–47) increasingly point to elite athletes being confronted not only with varied sports-specific, stress-triggering factors, but also with numerous stress sources outside of the elite sports environment including genetic, biological and environmental variables which may be associated with an increased vulnerability to mental health symptoms (6).

The fact that athletes are found to be subject to scrutiny from social media or coaches can take a toll on psychological wellbeing (31). External criticism from coaches in combination with internal pressure that elite athletes place on themselves have also been identified to affecting elite athletes' wellbeing and mental health (48).

## Interactions between stressors and resources in elite sport

Excellent health is essential for long-term, high-performance levels in sports. At the same time, an athlete's health is always at risk. In addition to the common stressors of life, elite athletes are confronted with numerous sports-specific stressors. From a salutogenetic perspective, elite athletes must successfully cope with the state of tension created by these stressors to generate health. Therefore, staying healthy represents a huge challenge.

According to the medical sociologist Aaron Antonovsky, health is considered as a position on a health ease/disease continuum and a movement in the direction towards health.

In Antonovsky's view everyone's resources are important factors in successfully responding to stressors and staying healthy (49).

Building on Balk's et al. (50) findings, it can be stated that with reference to the target group resources play a crucial role in promoting the wellbeing and mental health of athletes, especially when it comes to mitigate the impact of stress on elite athletes' wellbeing and mental health.

Balk et al. (50) found out that, physical resources such as recreational opportunities or training control can enhance the positive effects of sports-related stressors on physical strength. Emotional resources such as support from teammates or coaches can reduce stressors, such as conflicts or fear of failure. Cognitive Resources, i.e., access to information and control over training methods can help elite athletes improving decision-making and tactical execution.

According to Balk et al. (50) elite athletes should actively activate appropriate resources to cope with the stressors of elite sport, maintain wellbeing and mental health, and enhance performance, especially when used in a targeted and functional manner.

Therefore, the availability of sufficient resources is particularly important in demanding situations—which are found in the context of elite sports both in terms of the continuous delivery of peak performance and in terms of the double burden caused by a competitive sports career on the one hand and the completion of a school or academic career on the other hand (50).

In summary, stress impacts elite athletes' performance in both positive and negative ways, depending on their nature, intensity, and the availability of resources to buffer stress, enhance recovery, foster engagement, and prevent negative outcomes, ultimately supporting elite athletes' wellbeing and mental health.

Based on Balk's et al. research (50) some of these stress-influencing aspects on wellbeing and mental health are described below. The positive ones include:

• **Growth and development**: Moderate levels of physical, cognitive, and emotional stress can challenge athletes and promote growth, learning, and skill development. ​ For example, physical stressors help elite athletes build strength, endurance, and stamina, which are essential for peak performance.
• **Activation-enhancing effects:** When stress is coupled with sufficient resources, it can lead to positive outcomes such as increased creativity, learning, and optimal performance.

According to Balk's et al. findings (50) among the negative aspects, the following should be emphasised in particular:

• **Depletion of capacities:** Excessive physical, cognitive, or emotional stress can drain elite athletes' physical energy, mental focus, and emotional resilience, leading to reduced performance capabilities.
• **Increased risk of injury:** Physical stressors, especially when overwhelming, can increase the likelihood of injuries, further hindering performance.
• **Mental and emotional strain**: Cognitive and emotional stressors, such as decision-making under pressure or dealing with criticism, can lead to mental fatigue and emotional exhaustion, impairing focus and decision-making during competition.

As described above elite athletes face increasing physical, cognitive, and emotional stress that can either enhance or harm their wellbeing and mental health, depending on their intensity and whether elite athletes have access to adequate situational resources and coping strategies.

## Conclusion and future directions

Balancing stress with adequate resources (e.g., emotional support, autonomy) and coping strategies (e.g., detachment from sport-related activities) is crucial to ensure optimal wellbeing and mental health, to prevent burnout, and to support sustained high performance. Like physical health, mental health allows individuals to function, deal with stress, perform meaningful work, and contribute to society, (8).

Sports psychiatry, sport psychology, and clinical psychology offer an ecological perspective on the wellbeing and mental health of the athlete, addressing intra-individual (e.g., knowledge, attitudes, self-efficacy), inter-individual (e.g., connectedness and depth of social networks), organizational (e.g., organizational culture, organizational capacity, physical environment), and policy (e.g., informal rules, regulations) level factors that may influence mental health (12, 51).

To outline some future directions (see Table 1) it is recommended that sports psychiatry, sport psychology, and clinical psychology should work with and across individual, cultural, and environmental factors that affect the wellbeing and mental health of elite athletes (58).

**Table 1:** Future directions.

What is known	Knowledge gaps
Negative life event stress is a strong predictor for injury; injury has an impact on mental health issues (52–54), elite athletes operate in a highly complex social and organizational environment that mirrors the work context. This perspective allows the application of work and organizational psychology theories to understand and improve their well-being and mental health (50), sport-related stressors and resources consist of physical, cognitive, and emotional dimensions. Differentiating between these dimensions provides a better understanding of the specific challenges elite athletes face and the resources they need (50).	Which stressor predicts which outcome; situational aspects (e.g., dimensions) of stressors should be taken into consideration (e.g., frequency, intensity, duration) since their exclusion has been identified as a shortcoming of previous stress in sport research (55), a combined assessment of psychological stress and performance and mental health measures might be recommended for practical purposes (56), the impact of organizational stressors on mental health or psychological well-being has been “under-researched”; it is recommended to extend research on the outcomes and consequences of organizational stressors (57).

Sport organizations should optimize situational resources, monitor wellbeing and mental health, and incorporate recovery and coping strategies into training programs. Coaches and elite athletes should focus on activating matching resources and engaging in effective recovery activities.

The arguments mentioned above collectively emphasize the importance of addressing both stressors and resources to optimize the wellbeing and mental health of elite athletes.
